# Brain volumes in relation to loneliness and social competence in preadolescents born very preterm

**DOI:** 10.1002/brb3.1640

**Published:** 2020-04-24

**Authors:** Annika Lind, Susanna Salomäki, Riitta Parkkola, Leena Haataja, Päivi Rautava, Niina Junttila, Juha Koikkalainen, Jyrki Lötjönen, Virva Saunavaara, Riikka Korja, PIPARI Study Group

**Affiliations:** ^1^ Department of Psychology University of Turku Turku Finland; ^2^ Turku Institute for Advanced Studies (TIAS) University of Turku Turku Finland; ^3^ Department of Radiology University of Turku and Turku University Hospital Turku Finland; ^4^ Children's Hospital and Pediatric Research Center University of Helsinki and Helsinki University Hospital Helsinki Finland; ^5^ Public Health University of Turku and Clinical Research Centre of Turku University Hospital Turku Finland; ^6^ Department of Teacher Education University of Turku Turku Finland; ^7^ Finnish National Agency for Education Helsinki Finland; ^8^ Combinostics Ltd. Tampere Finland; ^9^ Division of Medical Imaging Department of Medical Physics Turku University Hospital Turku Finland; ^10^ Turku PET Center University of Turku and Turku University Hospital Turku Finland

**Keywords:** brain volumes, emotional loneliness, social competence, social loneliness, very preterm

## Abstract

**Introduction:**

The aim of the present study was to assess how regional brain volumes associate with self‐experienced social and emotional loneliness and social competence in very preterm and term‐born preadolescents.

**Materials and methods:**

Thirty‐four very preterm subjects (birthweight ≤1,500 g and/or gestational age <32 weeks) without neurodevelopmental impairments and/or major brain pathologies and 31 term‐born subjects underwent magnetic resonance imaging at 12 years of age. Regional brain volumes were measured using an automated image quantification tool. At 11 years of age, social and emotional loneliness were assessed with the Peer Network and Dyadic Loneliness Scale‐self‐report questionnaire and cooperating skills, empathy, impulsivity, and disruptiveness with the Multisource Assessment of Children's Social Competence Scale‐self‐report questionnaire.

**Results:**

In the very preterm group, a number of significant associations were found between smaller regional brain volumes and self‐experienced emotional loneliness, more impulsivity and more disruptiveness. In the control group, brain volumes and loneliness were not associated, and brain volumes and social competence were associated with a lesser degree than in the very preterm group.

**Conclusion:**

Experiences of emotional loneliness and poorer social competence appear to be more related to brain volumes in very preterm preadolescents than in those born full‐term. It also appears that in very preterm preadolescents, emotional loneliness may be more reflected in brain development than social loneliness.

## Graphical abstract

Due to the neurobiological vulnerability caused by a preterm birth, experiences of emotional loneliness and poorer social competence appear to be more related to brain volumes in very preterm preadolescents than in those born full‐term. It also appears that in very preterm preadolescents, emotional loneliness may be more reflected in brain development than social loneliness.

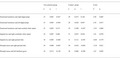

## 1 INTRODUCTION

Associations between difficulties in social skills and prematurity have been documented in several meta‐analyses (Arpi & Ferrari, 2013; Mathewson et al., 2017; Pyhala et al., 2017; Ritchie, Bora, & Woodward, 2015). Very preterm children tend to have poorer social skills and more peer problems and social withdrawal than children born at term (Ritchie et al., 2015). Extremely preterm subjects have been reported to have less friends, especially close ones, and to spend less time with their friends outside school at 12 years of age than very preterm and full‐term preadolescents (Ritchie, Bora, & Woodward, 2018). The difficulties in peer relationships have been shown to persist into adulthood in extremely preterm individuals (Linsell et al., 2019). However, very few studies have focused on the subjective experiences of loneliness and social competence of very preterm children. Loneliness is a subjective, distressing feeling of being without the kind of relationships the person desires (Weiss, 1973). Social loneliness refers to the absence of a social network or to the feeling that one is not part of a group, whereas emotional loneliness refers to the lack of close, intimate attachment to another person (Junttila, 2010). Social competence has been described as the ability to create and maintain social relationships (Anderson‐Butcher, Iachini, & Amorose, 2008), and it can be seen as a combination of prosocial behavior including cooperating skills and empathy and of refrainment from antisocial behavior including impulsive and disruptive behaviors (Junttila, Voeten, Kaukiainen, & Vauras, 2006).

Prematurity also increases the risk for suboptimal brain development, such as altered brain volumes. Brain volumes at term equivalent age have shown to predict developmental outcome, and volumes assessed at later ages have been shown to associate with various developmental domains, including some aspects of social competence, in preterm children and adolescents. Smaller hippocampal volume at term equivalent age has been reported to be associated with peer problems and poorer general social–emotional development at 5 years of age in girls born very preterm (Rogers et al., 2012). In 7‐ to 13‐year‐old very preterm subjects, reduced right medial prefrontal volume has been related to poorer socio‐behavioral abilities (Urbain, Sato, Hammill, Duerden, & Taylor, 2019). Increased gray matter volume in the fusiform gyrus has, in turn, been associated with social immaturity in very preterm adolescents (Healy et al., 2013), and smaller cerebellar volumes with lower psychosocial functioning at 15 and 19 years of age in very low‐birthweight subjects (Botellero et al., 2016).

The literature on associations between brain volumes and social competence in preterm subjects is scarce, and in the previous studies, social functioning has mostly been assessed using rather global measures. The aim of the present study was to assess how regional brain volumes associate with experienced loneliness and social competence in very preterm and term‐born subjects during early adolescence. Social loneliness, emotional loneliness, cooperating skills, empathy, impulsivity, and disruptiveness were analyzed separately. We hypothesized that due to the neurobiological vulnerability caused by a preterm birth, loneliness and social competence are more related to brain development in the very preterm group compared to the term‐born group.

## 2 METHODS

### 2.1 Participants

This study is part of the multidisciplinary PIPARI project (Development and Functioning of Very Low Birth Weight Infants from Infancy to School Age). All very low‐birthweight (≤1,500 g) infants born at Turku University Hospital in Finland between 2001 and 2006 who lived in the hospital catchment area and whose parents spoke and understood written Finnish or Swedish were eligible. From the beginning of 2004, the inclusion criteria were expanded to include all infants born <32 weeks of gestation, regardless of their birthweight. After exclusion of infants with major congenital anomalies or syndromes or chromosomal anomalies (*n* = 12), 228 were eligible and the parents of 219 infants agreed to participate in the main PIPARI Study. Participants born between May 2004 and December 2006 were invited to brain magnetic resonance imaging (MRI) at 12 years of age. Of the 91 eligible preadolescents, one could not be reached, 45 refused, and 45 agreed to take part. Subjects with known significant cognitive impairment (a full‐scale intelligence quotient <70), cerebral palsy, severe hearing impairment (loss of hearing requiring amplification in at least one ear), and/or visual impairment (binocular visual acuity <1.0 on the Snellen scale, high astigmatism of over 1 diopter or amblyopic eyes) were excluded from this substudy (*n* = 7). Of the 38 remaining preadolescents, 35 had questionnaire data available, and one was excluded due to a volume segmentation problem. This substudy thus includes 34 very preterm subjects. Three of the included subjects had no ophthalmological examination data available, but there were no blind subjects in the population. A control group of 31 term‐born subjects participating in the PIPARI project was included. The control group for the PIPARI Study was recruited 2001–2004 and consisted of healthy full‐term infants born at the same hospital. The parents of the first boy and the first girl born on each week were asked to take part in the study. If the parents refused, the next boy/girl was recruited. Inclusion criteria for the controls were a birthweight >−2.0 *SD* according to the age‐ and gender‐specific Finnish growth charts, and gestational age ≥37 weeks at birth, no admission to neonatal care during the first week of life, a Finnish speaking family, and a family residing inside the hospital catchment area. Control infants with a congenital anomaly or syndrome, or a self‐reported maternal use of illicit drugs or alcohol during the pregnancy were excluded. Control subjects born 2003–2004 were invited to the imaging at 12 years of age. Of 96 eligible, 39 chose to participate and 31 of these had both usable volumetric data and questionnaire data available.

The study has been approved by the Ethical Committee of the Hospital District of Southwest Finland. Informed consent was obtained from the parents, and at 12 years of age, the participants also gave their own written informed consent after receiving written information.

### 2.2 Brain imaging

A brain MRI was performed on the preterm infants with a 1.5 T Philips Intera scanner (Philips Medical Systems) at term equivalent age, and the findings are presented in Table 1. The findings were categorized into three groups to describe the level of brain pathology: (a) normal findings consisting of normal brain signal intensity and anatomy of the cortex and cortical gyration pattern, basal ganglia and thalami, posterior limb of internal capsule, white matter, germinal matrix, corpus callosum, cerebellum, pons and medulla oblongata, a width of extracerebral space of less than 5 mm, and a ventricular/brain ratio of less than 0.35; (b) minor pathologies consisting of the consequences of intraventricular hemorrhages of grades 1 and 2, such as minor linear T2 hyperintensities of the caudothalamic grooves or caudothalamic groove cysts smaller than 3 mm, a width of the extracerebral space of 5 mm, and a ventricular/brain ratio of 0.35; and (c) major pathologies consisting of the consequences of intraventricular hemorrhages of grades 3 and 4, cystic or cystic and hemorrhagic white matter lesions, as well as cystic or cystic and hemorrhagic lesions of the cortex, basal ganglia, thalamus, internal capsule, corpus callosum or cerebellum, focal T1 hyperintensities of deep or periventricular white matter corresponding to gliosis as well as increased width of extracerebral space by >5 mm, a ventricular/brain ratio of >0.35, ventriculitis, or focal infarctions.

**TABLE 1:** Background characteristics of the very preterm group and the control group

	Very preterm group *n* = 34	Control group *n* = 31
Gestational age, weeks, mean (*SD*) min–max	29 (3) 25–34	40 (2) 37–42
Birthweight, g, mean (*SD*) min–max	1,172 (322) 620–2,120	3,646 (471) 2,830–4,580
Boys/girls, *n* (%)	20 (59%)/14 (41%)	14 (45%)/17 (55%)
Birthweight *z*‐score, mean (*SD*) min–max	−1.4 (1.7) −4.4 to 2.2	0.0 (0.9) −1.6 to 2.2
Bronchopulmonary dysplasia, *n* (%)	5 (15%)	
Sepsis or meningitis, *n* (%)	1 (3%)	
Operated necrotizing enterocolitis, *n* (%)^a^	2 (6%)	
Retinopathy of prematurity, laser‐treated, *n* (%)^a^	1 (3%)	
Brain pathology at term equivalent age		
Normal, *n* (%)	27 (79%)	
Minor, *n* (%)	6 (18%)	
Major, *n* (%)	1 (3%)	
Brain pathology at 12 years of age		
Normal, *n* (%)	30 (88%)	31 (100%)
Minor, *n* (%)	4 (12%)	0
Major, *n *(%)	0	0

^a^ Data on necrotizing enterocolitis and retinopathy of prematurity were missing for two subjects.

A brain MRI was performed for the very preterm and control subjects at 12 years of age using 3 T Philips Ingenuity TF PET/MR (Philips). Mean age at assessment of the very preterm subjects was 12.8 (*SD* 0.5, min 12.1, max 13.8) and the control subjects 12.8 (*SD* 0.3 min 12.1, max 13.4). Three imaging protocols were used for the clinical assessment: a T2‐weighted set of axial slices with a 4.82‐s repetition time (TR) and an 80‐ms echo time (TE) and 3 mm slice thickness; a coronal fluid attenuation inversion recovery sagittal images with a 10‐s TR, a 2.8‐s inversion time, a TE of 125 ms, a slice thickness of 4 mm; and a 3D T1 set of sagittal slices with a 8.1‐ms TR, a 3.7‐ms TE, and isotropic 1 mm voxel. The magnetic resonance images were assessed by a neuroradiologist with 25 years of experience in pediatric neuroradiology. The MRI findings at the age of twelve were also categorized into three groups (Table 1): (a) Normal findings consisted of normal brain signal intensity and anatomy of the cortex and cortical gyration pattern, basal ganglia and thalami, internal and external capsule, white matter, corpus callosum, cerebellum, pons and medulla oblongata, and normal cerebrospinal fluid spaces; (b) minor pathologies such as mild prominence of one of the four lateral ventricular horns without brain parenchyma pathologies or minor punctuate cerebral white matter T1 hyperintensity; and (c) major pathologies consisted of T2 hyperintensity of the cerebral or cerebellar parenchyma corresponding to focal hemosiderin collection, symmetric or asymmetric white matter damage corresponding to white matter gliosis, marked dilatation of the ventricles or marked dilatation of the cortical cerebrospinal fluid spaces or signs of infarcts or cystic and/or hemorrhagic white or gray matter damage.

The volumes of the brain structures were measured from T1 images using an automated image quantification tool (Combinostics Ltd., www.cneuro.com/cmri/), which divides the brain into 133 regions using a multi‐atlas method. First, the 28 best‐matching atlases were selected from the original 79 manually segmented atlases (http://www.neuromorphometrics.com/), and the selected atlases were nonrigidly registered with the T1 image. The brain segmentation was generated from the 28 atlas segmentations using the expectation–maximization algorithm. The following volumes were included in the analyses: amygdala (right and left), caudate (right and left), cerebellum exterior (right and left), cerebellum white matter (right and left), cerebellar vermal lobules I–V, cerebellar vermal lobules VI–VII, cerebellar vermal lobules VIII–X, hippocampus (right and left), pallidum (right and left), putamen (right and left), thalamus proper (right and left), fusiform gyrus (right and left), total cerebral white matter, frontal lobe (right and left), temporal lobe (right and left), parietal lobe (right and left), and occipital lobe (right and left).

### 2.3 Assessment of loneliness and social competence

The loneliness and social competence of the very preterm subjects and the control subjects were assessed with the Peer Network and Dyadic Loneliness Scale (PNDLS; Hoza, Bukowski, & Beery, 2000; Junttila & Vauras, 2009) and the Multisource Assessment of Children's Social Competence Scale (MACSCS; Junttila et al., 2006; Kaukiainen, 2005) questionnaires the year the subjects turned 11. The very preterm participants filled in the questionnaires during a psychological assessment, which was part of the developmental follow‐up in PIPARI Study. The controls completed the questionnaires at home. The PNDLS is a self‐report questionnaire containing five questions in the form of paired statements concerning social loneliness and five concerning emotional loneliness. The sum scores for social loneliness and emotional loneliness both range from five to twenty, and higher scores reflect more loneliness. MACSCS is a self‐report questionnaire assessing cooperating skills, empathy, impulsivity, and disruptiveness based on a total of 15 questions. Weighted sum scores are created from the raw scores according to the manual (Kaukiainen, 2005), and higher scores reflect more of the assessed trait. Weighted sum scores for cooperating skills range from 3.22 to 12.88, for empathy from 1.76 to 7.04, for impulsivity from 2.23 to 8.92, and for disruptiveness from 2.63 to 10.52.

### 2.4 Statistical analyses

The statistical analyses were performed using the SPSS 24.0 software (IBM, Armonk, NY, USA). For the analyses, the raw volumes were scaled based on total intracranial volume. The PNDLS and MACSCS scores were used as continuous variables. For group comparisons, the *t* test for independent samples or the Mann–Whitney U test was used, as appropriate. Associations between the scaled volumes and questionnaire scores were analyzed using Spearman's rank correlation test. For the significant associations, the impact of gender and age at MRI assessment was analyzed with a partial Spearman's rank correlation test. The associations reported in the Results section are based on the partial Spearman's rank correlation test and are thus controlled for gender and age at MRI. The significance of the difference between the correlation coefficients in the very preterm group and the control group was calculated with *z* test (Fisher's *r*‐to‐*z* transformation). *p*‐values <.01 were considered statistically significant.

## 3 RESULTS

The neonatal characteristics and brain pathology at term equivalent age and at 12 years of age are shown in Table 1. None of the very preterm or the control children had major brain pathologies at 12 years of age. The scaled regional brain volumes are presented in Table 2 and mean scores of PNDLS and MACSCS in Table 3. The volumes did not differ significantly (*p* < .01) between the very preterm and the control groups. The mean scores of PNDLS and MACSCS indicated more experienced loneliness and poorer social competence in the very preterm group, but the differences were not significant. The PNDLS and MACSCS scores, birthweight, and gestational age of the PIPARI Study subjects not included in the current study are presented in Table 4. These variables did not differ significantly between included and not included very preterm subjects, or between included and not included control subjects.

**TABLE 2:** Regional brain volumes (ml), scaled for total intracranial volume, in the very preterm group and the control group

	Very preterm group, *n* = 34 mean (*SD*) median, min–max	Control group, *n* = 31 mean (*SD*) median, min–max
Amygdala, right	1.2 (0.1) 1.2, 0.9–1.5	1.2 (0.1) 1.2, 0.9–1.3
Amygdala, left	1.2 (0.1) 1.2, 1.0–1.4	1.2 (0.1) 1.2, 0.9–1.3
Caudate, right	3.7 (0.4) 3.8, 2.8–4.3	3.8 (0.3) 3.7, 3.3–4.7
Caudate, left	3.6 (0.5) 3.6, 2.5–4.3	3.7 (0.4) 3.6, 3.2–4.8
Cerebellum exterior, right	55.1 (3.5) 54.9, 49.3–62.5	54.6 (3.8) 53.9, 47.3–62.7
Cerebellum exterior, left	54.8 (3.9) 55.4, 46.1–61.5	54.0 (3.8) 54.1, 48.0–62.2
Cerebellum white matter, right	12.0 (1.3) 12.1, 9.9–15.1	12.7 (1.1) 12.6, 10.1–14.6
Cerebellum white matter, left	11.9 (1.2) 11.9, 9.7–14.5	12.5 (1.0) 12.6, 9.5–14.0
Cerebellar vermal lobules I–V	4.8 (0.7) 4.9, 2.6–6.0	4.9 (0.5) 4.9, 3.7–5.7
Cerebellar vermal lobules VI–VII	2.1 (0.3) 2.1, 1.5–2.8	2.1 (0.3) 2.1, 1.5–2.7
Cerebellar vermal lobules VIII–X	3.0 (0.4) 3.0, 2.3–3.9	2.9 (0.3) 3.0, 2.1–3.7
Hippocampus, right	3.7 (0.3) 3.7, 3.1–4.3	3.9 (0.3) 3.8, 3.4–4.4
Hippocampus, left	3.6 (0.3) 3.6, 3.0–4.4	3.7 (0.3) 3.7, 3.2–4.3
Pallidum, right	1.3 (0.2) 1.3, 1.0–1.7	1.4 (0.1) 1.4, 1.1–1.7
Pallidum, left	1.3 (0.2) 1.3, 0.9–1.6	1.3 (0.1) 1.4, 1.0–1.6
Putamen, right	5.1 (0.6) 5.0, 4.1–6.0	5.0 (0.5) 5.0, 4.3–6.0
Putamen, left	5.2 (0.6) 5.1, 4.0–6.2	5.3 (0.5) 5.3, 4.4–6.1
Thalamus proper, right	8.4 (0.4) 8.4, 7.6–9.5	8.4 (0.3) 8.4, 7.8–9.1
Thalamus proper, left	8.7 (0.5) 8.8, 7.5–10.0	8.8 (0.4) 8.8, 8.2–9.4
Fusiform gyrus, right	9.4 (1.0) 9.3, 7.8–11.6	9.0 (0.8) 8.8, 7.1–10.6
Fusiform gyrus, left	9.2 (0.8) 9.4, 7.2–10.6	9.0 (0.9) 8.9, 6.8–11.2
Cerebral white matter, total	394.4 (20.9) 395.4, 352.5–444.4	397.9 (14.0) 397.0, 366.4–426.4
Frontal lobe, right	128.3 (6.6) 129.1, 112.5–145.0	130.3 (6.4) 129.5, 120.5–144.8
Frontal lobe, left	127.6 (6.5) 127.7, 111.4–142.3	129.7 (6.3) 129.9, 118.1–143.2
Temporal lobe, right	72.1 (3.0) 72.5, 65.6–77.4	72.8 (3.1) 73.7, 63.1–79.0
Temporal lobe, left	72.6 (3.1) 72.7, 64.9–79.2	72.5 (3.0) 72.6, 61.7–76.4
Parietal lobe, right	69.4 (3.9) 69.8, 61.1–79.5	69.8 (3.7) 70.0, 62.3–78.6
Parietal lobe, left	70.1 (3.7) 70.2, 60.0–78.5	69.7 (4.9) 70.6, 57.4–77.0
Occipital lobe, right	44.3 (3.2) 44.0, 36.7–51.3	42.9 (2.8) 42.3, 38.4–48.6
Occipital lobe, left	42.9 (2.9) 42.8, 36.7–50.3	42.0 (2.9) 42.2, 36.0–48.6

**TABLE 3:** Loneliness and social competence scores in the very preterm group and the control group. Higher scores indicate more of the assessed experience or trait

	Very preterm group, *n* = 34 mean (*SD*) median, min–max	Control group, *n* = 31 mean (*SD*) median, min–max
Social loneliness^a^	7.7 (2.0) 7.0, 5.0–11.0	7.3 (2.5) 6.5, 5.0–13.0
Emotional loneliness^a^	7.3 (2.1) 7.0, 5.0–14.0	6.8 (2.1) 6.0, 5.0–13.0
Cooperating skills	10.0 (1.3) 10.0, 7.1–12.3	10.8 (1.4) 10.9, 8.4–12.9
Empathy	6.0 (0.7) 5.9, 4.7–7.0	6.3 (0.7) 6.4, 5.3–7.0
Impulsivity	4.2 (1.2) 4.5, 2.2–6.8	4.2 (1.7) 3.7, 2.2–8.9
Disruptiveness^a^	3.8 (0.9) 3.3, 2.7–5.9	3.7 (1.0) 3.6, 2.7–6.4

^a^ Data on social and emotional loneliness were missing for one preterm subject. Data on social loneliness were missing for one control subject, data on emotional loneliness for two, and data on disruptiveness for one.

**TABLE 4:** Loneliness and social competence scores, birthweight, and gestational age of those very preterm and control subjects of the PIPARI Study Group who were not included in the present study

	Very preterm subjects, *n* = 138 mean (*SD*) median, min–max	Control subjects, *n* = 105 mean (*SD*) median, min–max
Social loneliness^a^	8.0 (2.5) 8.0, 5.0–15.0	7.4 (2.3) 7.0, 5.0–14.0
Emotional loneliness^a^	7.8 (2.6) 8.0, 5.0–17.0	7.7 (2.7) 7.0, 5.0–16.0
Cooperating skills^a^	10.2 (1.3) 10.2, 6.4–12.9	10.2 (1.3) 10.3, 6.4–12.9
Empathy^a^	6.0 (0.9) 5.9, 1.8–7.0	6.0 (0.7) 5.9, 4.1–7.0
Impulsivity^a^	4.1 (1.3) 4.4, 2.2–8.9	4.3 (1.3) 4.5, 2.2–8.2
Disruptiveness^a^	3.7 (1.0) 3.4, 2.7–7.1	4.1 (1.1) 4.0, 2.7–8.0
Birthweight, g	1,103 (317) 1,113, 400–2,025	3,636 (413) 3,660, 2,570–4,810
Gestational age, weeks^a^	29 (3) 29, 23–36	40 (1) 40, 37–42

^a^ Data on social and emotional loneliness were missing for seven preterm subjects, on cooperating skills for six, on empathy for one, on impulsivity for two, and on disruptiveness for three. Data on social loneliness were missing for three control subjects, on emotional loneliness for five, on impulsivity for three, on disruptiveness for one, and on gestational age for one.

Analyses of associations between the scaled volumes and PNDLS and MACSCS scores, controlled for gender and age at assessment, showed several significant associations in the very preterm group. Emotional loneliness was significantly associated with smaller volumes of the right (*p* = .001, *r* = −.567) and left (*p* = .005, *r* = −.494) hippocampi and with a larger volume of cerebral total white matter (*p* = .003, *r* = .515). Impulsivity was significantly associated with a larger volume of right cerebellar white matter (*p* = .007, *r* = .468) and with a smaller volume of right parietal lobe (*p* = .005, *r* = −.488). In addition, disruptiveness was significantly associated with a smaller volume of the right parietal lobe (*p* = .000, *r* = −.605). In the control group, disruptiveness was significantly associated with a smaller volume of the left fusiform gyrus (*p* = .006, *r* = −.508). No significant associations with any other scales appeared in the control group. Comparison of the correlation coefficients in the very preterm group and the control group (Table 5) showed significant differences for the association between emotional loneliness and the volume of right hippocampi (*Z* = 2.94, *p* = .003), and between emotional loneliness and the volume of total cerebral white matter (*Z* = −2.91, *p* = .004).

**TABLE 5:** Differences between the correlation coefficients in the very preterm and the control groups

	Very preterm group			Control group			*Z* test	
	*n*	*p*	*r*	*n*	*p*	*r*	*Z*	*p*
Emotional loneliness and right hippocampi	33	.001	−.567	29	.473	.144	2.94	.003
Emotional loneliness and left hippocampi	33	.005	−.494	29	.630	.097	2.38	.017
Emotional loneliness and total cerebral white matter	33	.003	.515	29	.300	−.207	−2.91	.004
Impulsivity and right cerebellar white matter	34	.007	.468	31	.853	.036	1.81	.070
Impulsivity and right parietal lobe	34	.005	−.488	31	.121	−.295	−0.88	.379
Disruptiveness and right parietal lobe	34	.000	−.605	30	.634	−.094	−2.30	.021
Disruptiveness and left fusiform gyrus	34	.513	−.120	30	.006	−.508	1.67	.095

## 4 DISCUSSION

The focus of this study was to assess whether regional brain volumes are associated with experienced loneliness and social competence in very preterm and term‐born preadolescents. The study groups were well defined, subjects with neurodevelopmental impairments and major brain pathologies were excluded, and total intracranial volume, gender, and age at assessment were taken into account in the analyses. Still, regional brain volumes were found to be associated with emotional loneliness, impulsivity, and disruptiveness in the very preterm group during early adolescence. In addition, as hypothesized, brain volumes and social competence were associated with a lesser degree in the control group than in the preterm group.

The literature on associations between brain volumes and social competence in preterm subjects is limited and not fully comparable with the present study due to differing methods and ages at the time of assessment. However, smaller hippocampal volumes at term equivalent age have been shown to be predictive of peer problems and poorer socio‐emotional development at 5 years of age in girls born very preterm (Rogers et al., 2012). Our study showed an association between smaller hippocampal volumes and more experiences of emotional loneliness also later in childhood. The associations between a larger total cerebral white matter volume and more experiences of emotional loneliness, and between a larger volume of right cerebellar white matter and impulsivity, were unexpected. These findings are, however, interesting in relation to a review which proposed that subjects with autism spectrum disorder have increased whole brain volume (Pagnozzi, Conti, Calderoni, Fripp, & Rose, 2018). In addition, increased cerebellar volume has appeared to be associated with autism spectrum disorder in a meta‐analysis (Traut et al., 2018). A smaller volume of right parietal lobe was in our study associated with more impulsivity and disruptiveness, which is well in line with a study by Botellero et al. (2017). They found that a smaller volume of the parietal cortex was associated with more inattention and hyperactivity and with poorer psychosocial functioning in adolescents born with a very low birthweight. As for the control group of the present study, a smaller volume of left fusiform gyrus was associated with more disruptiveness. A smaller left fusiform gyrus has previously been associated with conduct problems in youths (Rogers & Brito, 2016), but the direction appears reversed in very preterm adolescents in whom increased gray matter volume of the fusiform gyrus has been associated with social immaturity (Healy et al., 2013).

On the whole, our findings support studies presenting associations between brain volume and social competence in very preterm subjects. We are, however, aware that socio‐emotional problems in preterm children result from an interplay between various factors, such as biological vulnerabilities, early life stress, parental behavior, cognitive impairments, and environmental factors (Montagna & Nosarti, 2016). Also, it is not possible to determine the causal relationship between brain volumes and loneliness or social competence based on our study. The above‐referred findings of associations between smaller hippocampal volumes at term equivalent age and later peer problems and poorer socio‐emotional development (Rogers et al., 2012) indicate that suboptimal brain development and growth may underlie poorer social competence in preterm children. But the possibility that experiences relating to social situations and loneliness could have affected the brain volumes should not be omitted (Cacioppo, Capitanio, & Cacioppo, 2014). It must be noted that experienced loneliness and social competence in our study were assessed the year the participants turned 11, while the MRI was performed at 12 years of age.

Interestingly, no significant associations between social loneliness and brain volumes appeared in our study, while emotional loneliness was associated with smaller hippocampal volumes and with a larger volume of total cerebral white matter. The experiences and consequences of social loneliness and emotional loneliness differ from each other (Salo, Junttila, & Vauras, 2019). Compared to social loneliness, emotional loneliness has been shown to relate more to factors of individual psychological well‐being, such as emotional problems (Qualter & Munn, 2002), poorer individual life satisfaction (Salimi, 2011), and even mortality (O'Suilleabhain, Gallagher, & Steptoe, 2019). In the study of Qualter and Munn (2002), children who experienced emotional loneliness had, for example, low self‐worth and inaccurate perceptions of self and relationships compared to the socially lonely children. Accordingly, as emotional loneliness appears to be a more significant risk factor for poorer psychological well‐being than social loneliness, it may be speculated that this is reflected in our results of more associations between brain volumes and emotional loneliness. Emotional loneliness can be accompanied by dysfunctional emotional regulation, such as depression and anxiety. Findings about the relation between brain structure and these conditions are mixed, but, for instance, reduced hippocampal volume, has in some studies been suggested to be associated with depression or anxiety (Barch et al., 2019; Merz, He, & Noble, 2018; Moon & Jeong, 2017).

One limitation of the present study is small group sizes. However, we have not found previous studies on associations between brain volumes and social or emotional loneliness in preterm subjects, and our study provides novel findings about the neural correlates of social functioning including loneliness. Another limitation is that a self‐report questionnaire can give a view of the subjects' social competence that is more positive or negative than the views of others (Kaukiainen, 2005). It would have been optimal to also obtain a perspective on the subjects' social competence from peers, parents, and teachers, even though loneliness is a very subjective emotion. A further restraint is differing ages at MRI and at assessment of loneliness and social competence. Children's loneliness have, however, been shown to be stable (Junttila & Vauras, 2009; Salo et al., 2019), and peer relationship problems have been shown to persist through childhood and adolescence in extremely preterm subjects (Linsell et al., 2019). The multi‐atlas segmentation method has mainly been developed for the segmentation of adult brains. Consequently, the segmentation accuracy of the child brain may be lower than the accuracy of segmentation of the adult brain. However, this possible effect does not differ between the very preterm group and the control group, and thus does not cause bias in the results.

## 5 CONCLUSION

Brain volumes were in this study associated with emotional loneliness, impulsivity, and disruptiveness in very preterm preadolescents. The brain volumes and loneliness were not associated in the full‐term group, and the volumes and social competence were also associated with a lesser degree than in the very preterm group. Experiences of emotional loneliness and poorer social competence thus appear to be more related to brain development in very preterm preadolescents than in those full‐term, though in interplay with other factors. In healthy full‐term children, social development may possibly be mainly related to their contextual factors such as family environment. It also appears that in very preterm subjects during early adolescence, emotional loneliness may be more strongly reflected in brain development than social loneliness. Comparable studies with larger study groups, focusing especially on the differences between social and emotional loneliness, are needed.

## CONFLICT OF INTEREST

None declared.

## AUTHOR CONTRIBUTION

All authors contributed to the study conception or design and to the interpretation of the results. All authors read and revised the manuscript and approved the final manuscript to be submitted.

## Data Availability

Research data are not shared.
